# P231: Risk factors for Acinetobacter baumannii colonization and infection among patients admitted to intensive care units

**DOI:** 10.1186/2047-2994-2-S1-P231

**Published:** 2013-06-20

**Authors:** M Giannella, S Di Bella, G D'Este, ME Halgass, A Lappa, MG Tolusso, E Ferrari, M Tronci, E Grilli, N Corradetti, N Petrosillo

**Affiliations:** 1 2nd Division of Infectious Diseases, National Institute of Infectious Diseases "Lazzaro Spallanzani", Italy; 2 Intensive Care Unit, Policlinico Casilino, Italy; 3 Microbiology, Policlinico Casilino, Italy; 4 Cardiosurgical Intensive Care Unit, Ospedale San Camillo, Italy; 5 Intensive Care Unit, Ospedale San Camillo, Italy; 6 Neurosurgical Intensive Care Unit , Ospedale San Camillo, Italy; 7 Microbiology, Ospedale San Camillo, Italy; 8 Microbiology, Aurelia Hospital, Roma, Italy

## Introduction

Active surveillance cultures (SCs) for *Acinetobacter baumannii* (AB) has been suggested as a strategy to control AB spread in Intensive Care Units (ICUs). However, standardized screening shemes and understanding of which patients and body sites are most commonly colonized by AB are lacking.

## Objectives

To identify risk factors for AB colonization upon ICU admission.

## Methods

Multicenter prospective study of all patients admitted for ≥ 48 hours to 6 ICUs, in Rome. SCs included rectal swab (RS) and pharyngeal swab (PS) or tracheal aspirate (TA) in patients on mechanical ventilation. SCs were taken on ICU admission and once weekly until discharge from ICU.

## Results

From May to Sept 2012, 847 patients were admitted to the 6 ICUs; 261 remained for ≥ 48 hours and 201 were screened. Overall, 359 RS, 270 TA and 264 PS were taken and AB was isolated in 5.6%, 8.1% and 1.1% of them, respectively, with a total of 53 AB. All the strains were resistant to carbapenems. Of the 201 screened patients, 59.2% were male, the median age was 65 years (IQR 50-75), median Charlson and APACHE II scores were 5 (IQR 3-6) and 13 (IQR 9-17), respectively. Commonest causes for ICU admission were respiratory failure (32.3%) and post surgery (30.2%). Fourteen patients (7%) were found to be AB colonized at ICU admission, whereas 9 (4.5%) became colonized during ICU stay within a median of 7 days (IQR 6-10). Significant differences between AB colonized and non-colonized patients at ICU admission were found for septic shock (21.4% vs. 4.3%) and prior antibiotic therapy (78.6% vs. 43.3%).

## Conclusion

Our preliminary analysis showed that prior antibiotic therapy and septic shock were significantly associated with AB colonization at ICU admission.

## Disclosure of interest

None declared

